# Obituary

**Published:** 1912-02-03

**Authors:** 


### Obituary.

Dr. Charles Warden has died, at the age of eighty-four, at his residence in Weston-super-Mare. As a student of St. George's Hospital he qualified in 1850 as M.R.C.S. Eng., and took the M.D. of King's College, Aberdeen, the following year. Dr. Warden associated himself very soon with ear and throat work, and was elected honorary surgeon to the Birmingham and Midland Ear and Throat Hospital, the Birmingham Royal Orthopædic and Spinal Hospital, and the Birmingham Royal Deaf and Dumb Institute. Not only had he a large practice in Birmingham, but he also took a prominent part in the philanthropic work and civic life of the city. He was at one time a justice of the peace for the city, and president of the British Benevolent Society in 1888. Numerous societies connected with his specialty can claim him as past president or vice-president. Some ten years ago he retired to Weston-super-Mare to enjoy a well-earned rest from all the activities inseparable from his great professional and municipal interests.

Dr. Charles Alfred Lee, of Hull, has died at the age of eighty-six. He was not only one of the most successful practitioners of Hull, but also one of its most prominent benefactors. No one but himself could accurately gauge the extent of his liberal donations to charities. To the Royal Infirmary at Hull he was exceedingly generous, and one of the wards there is named after him. He left £150,000 to the city for the erection of almshouses, besides other large bequests for benevolent purposes. Dr. Lee qualified in 1848 as M.R.C.S. Eng., and became M.D. of King's College, Aberdeen, in 1859. He was a great lover of horses, and in the past was a keen follower of the hounds.

Dr. Reginald Gershom Sparkes, house physician at St. Mary's Hospital, has met an untimely death from meningitis after a short illness. Dr. Sparkes only qualified last year, and had not long been in residence at his own hospital before he contracted his fatal illness.

Dr. J. Waddell Jeffreys Oswald has died, at his residence in London, S.W., at the age of eighty. Dr. Oswald was surgeon to the South Metropolitan Gasworks, and late medical officer to the Royal South London Dispensary. He was a member of the council of the British Medical Association, and a former vice-president of the local branch. Dr. Oswald studied at Anderson's College, Glasgow, and at the University of Edinburgh; he qualified in 1862, subsequently taking several other degrees, including the M.D. St. Andrew's and the fellowship of the Royal College of Surgeons, Edinburgh.

Dr. Chas. Dudley Garrett has died at Faversham at the age of forty-five. He was a student of Westminster Hospital, and qualified in 1894 at the Conjoint Board. He was formerly in practice at Dewsbury, where he held the appointment of police surgeon, and where he devoted a great deal of attention to the question of anthrax infection among the rag-sorters in the mills. A few years ago he transferred himself to Faversham, and was elected surgeon to the local cottage hospital. Dr. Garrett for many years took a very active interest in ambulance work, and possessed the long-service medal. He was an honorary Serving Brother of the Association of St. John of Jerusalem, and in 1900 was made an honorary life member of the St. John Ambulance Association in recognition of his services as examiner and lecturer. The Association was well represented at the funeral at Faversham.

Dr. Wm. Dashwood Kingdon, who has just died at his residence in Exeter, at the age of ninety-three, was probably the holder of a record for the number of years on the medical register, for he qualified in 1837 at the University of Edinburgh as M.D., and in the same year became a Licentiate of the Royal College of Physicians, also of Edinburgh. Dr. Kingdon was formerly physician to the Exeter Dispensary, and Resident Medical Superintendent of St. Thomas's Lunatic Hospital, Exeter.

